# Listen to your inner body: embodied emotions in predictive neuroscience and traditional East Asian medicine

**DOI:** 10.3389/fnhum.2026.1807034

**Published:** 2026-04-20

**Authors:** Seoyoung Lee, Seok-In Yoon, Hsien-Yin Liao, Jong Woo Kim, In-Seon Lee, Younbyoung Chae

**Affiliations:** 1 Department of Behavioral Medicine, Faculty of Medicine, Institute of Basic Medical Sciences, University of Oslo, Oslo, Norway; 2 Department of Neuropsychiatry, College of Korean Medicine, Kyung Hee University, Seoul, Republic of Korea; 3 School of Post-Baccalaureate Chinese Medicine, College of Chinese Medicine, China Medical University, Taichung, Taiwan; 4 Department of Meridian and Acupoints, College of Korean Medicine, Kyung Hee University, Seoul, Republic of Korea

**Keywords:** acupuncture, allostasis, emotion, interoception, predictive processing, Qi

## Abstract

The self is increasingly conceptualized as an embodied, predictive process in contemporary cognitive and affective neuroscience. The brain continually infers the causes of both exteroceptive and interoceptive signals in order to minimize prediction error and maintain allostatic balance. Within this framework, emotion can be understood as an inference about changes in bodily states. Parallel themes have long been articulated in traditional East Asian medicine (TEAM), where emotions are understood to modulate the flow of Qi and clinical practice is aimed at restoring dynamic balance through breath, movement, attention, acupuncture, and related interventions. This study brings these traditions into dialogue, arguing that the phenomenology of bodily patterns and the directional qualities of emotion in TEAM are compatible with predictive-processing accounts of interoception, allostasis, and affect regulation. We summarize key research on autonomic flexibility, emotional “body maps,” and interoception; integrate clinical observations from acupuncture practice; offer testable cross-framework hypotheses; and outline implications for mental health and wellbeing. We advocate a pluralistic, pragmatic approach that acknowledges conceptual diversity while using points of convergence to guide research and practice, rather than forcing one framework into the terms of the other. Bridging modern neuroscience with traditional insights can support a more deeply embodied understanding of the self and provide new avenues for investigating the regulation of emotional life.

## 1 Introduction

In the West, a long-standing philosophical stance views cognition as the hallmark of personhood, encapsulated in Descartes’ “I think, therefore I am.” This view has been challenged by recent developments in neuroscience, which emphasize selfhood as an embodied, temporally extended process (Clark, 2013; Craig, 2002; Seth and Critchley, 2013). The brain creates generative simulations based on prior experiences. Predictive processing theory characterizes the brain as an inferential organ, maintaining generative models that anticipate the hidden causes of sensory inputs and act to reduce prediction errors through perceptual updating and autonomic/allostatic regulation (Friston, 2010; Sterling, 2012). Within this framework, perception is a form of “controlled hallucination” disciplined by incoming evidence, and emotion is a perception of bodily change, an inference about interoceptive fluctuations given context and prior beliefs (Barrett, 2017; Seth and Friston, 2016). The brain continuously predicts what is likely to occur both inside the body and outside of it. Emotions are thus more than simply thoughts; they can be understood as perceptions of changes in bodily states.

According to traditional East Asian medicine (TEAM), emotions are directly related to the dynamics of the body (Ots, 1990). Within this tradition, Qi broadly refers to the dynamic functional activity that sustains physiological and experiential processes in the body, rather than a single material substance or directly measurable physiological variable. Somatic processes are understood to participate in the construction of emotions and emotion-related disorders, which are linked to certain subsystems, often indexed by organs such as the heart or liver (Scheid, 2013). Organ-based metaphors that suggest interior physical imagery are figurative yet appear to be grounded in systemic bodily and psychological regularities rather than being arbitrary. In this view, emotions are deeply grounded in embodied experience, and are characterized in terms of their relations to distinct visceral systems (Lee et al., 2017). According to classical sources, emotions are often described as directional influences that shape or modulate the flow of Qi throughout functional networks (which are not directly equivalent to biomedical organ systems). Therapeutic practices such as acupuncture, meditation, Qigong, and dietary control are aimed at restoring disrupted flow patterns. Despite their different terminologies, TEAM and modern neuroscientific explanations converge on a fundamental idea: paying attention to the predictable interaction between the body and brain is essential for understanding and effectively modifying lived experience. Recent conceptual analyses have likewise explored parallels between predictive neuroscience, allostasis, and traditional Chinese medicine frameworks, suggesting functional analogies between predictive regulatory processes and classical concepts such as Qi and systemic balance (Minelli, 2026).

Understanding ourselves through TEAM presents a fascinating and challenging inquiry at the crossroads of medicine, cognitive science, and philosophy. In this article, we discuss points of convergence and complementarity between these frameworks, propose a translational research agenda, and sketch practical implications for clinical care. We make three main contributions. First, we articulate explicit conceptual bridges between the directional and flow-based accounts of emotion in TEAM and Western predictive-processing models of interoception, allostasis, and affect regulation. Second, we integrate evidence on autonomic flexibility, bodily maps of emotion, and interoceptive processing with clinical observations from acupuncture practice to generate operational hypotheses. Third, we outline a translational research agenda that uses points of convergence and divergence between TEAM and contemporary neuroscience to guide experimental design and clinical trials, rather than attempting to assimilate one framework into the other.

## 2 Theoretical frameworks and cross-cultural concepts of embodied emotions

### 2.1 Neuroscience perspective: predictive brains and interoceptive inference

According to theories of predictive processing, the brain maintains hierarchical generative models that forecast sensory inputs; discrepancies (prediction errors) are resolved by updating posterior beliefs or by acting to change sensory inputs, including through autonomic adjustments that regulate the internal milieu (Clark, 2013; Friston, 2010; Sterling, 2012). A critical component of this architecture is interoception, or the ability of the nervous system to detect, interpret, and integrate internal bodily signals (cardiovascular, respiratory, gastrointestinal, and immune signals), thereby enabling continuous mapping of the body’s internal landscape across both conscious and unconscious levels (Craig, 2002; Khalsa et al., 2018). Autonomic responses, affective experience, and metabolic demand are all connected by interoceptive inference (Seth and Friston, 2016; Barrett and Simmons, 2015). On this account, humans experience moments of intense interoceptive sensations as emotions. Building on early insights from James–Lange theory, which emphasized the importance of bodily feedback in emotion, modern predictive processing views emotion as the brain’s best estimate of the causes and consequences of interoceptive fluctuations based on prior expectations and contextual signals rather than a detached label imposed on unprocessed sensation (Khalsa et al., 2018; Barrett and Simmons, 2015).

Neural foundations of interoceptive inference, including the posterior and anterior insula, anterior cingulate, somatosensory cortices, and brainstem nuclei, integrate visceral afferents with higher-order appraisals of contextualized concepts and goals (Craig, 2002; Barrett, 2017). The sense of an embodied, predictive self emerges from this phenomenological stability, which in turn depends on the calibration of interoceptive priors and the flexibility of autonomic control. The brain’s model of the body is continuously updated by the interoceptive networks that generate predictions about bodily states and compare them with incoming sensory signals. Indeed, experimental manipulations that change interoceptive signaling (for example, breathing patterns that increase vagal tone) can shift affective experiences and threat assessments, demonstrating the role of interoception in reshaping emotion (Critchley and Harrison, 2013; Laborde et al., 2017).

Affective traits and vulnerabilities, such as anxiety and panic, are predicted by individual differences in interoceptive accuracy (objective performance), sensitivity (subjective beliefs), and awareness (metacognitive correspondence between the two) (Garfinkel et al., 2015). Thus, interoception provides the basis for embodied emotions, as the brain’s predictive model continuously interprets and regulates internal signals via detection, appraisal, coordination, and regulatory control (Chen et al., 2021). Recently, a research model has been proposed that positions interoception as a central mechanism in whole-person health (Chen and Langevin, 2025).

### 2.2 Traditional East Asian medicine perspective: direction, flow, balance

TEAM conceptualizes the body as a network of interdependent functional systems whose activities are organized through dynamic circulation and transformation. The organ systems (*zang-fu*) represent coordinated functional constellations across tissues, emotions, and behaviors rather than discrete anatomical structures (Lee et al., 2017). According to this perspective, health is a continuous state of balance (characterized by adaptive variability both within and between systems) rather than a fixed moment in time, and emotions are embodied processes that arise from these functional networks (Ots, 1990; Scheid, 2013). Emotions are conceptualized as directional and qualitative influences on Qi: anger drives upward movement, fear induces downward movement, worry engenders knotting and stagnation, and joy promotes dispersion. Across mind–body interventions such as acupuncture, meditation, and Qigong, practitioners frequently report complex bodily sensations that are interpreted as therapeutically meaningful (Beissner, 2020).

TEAM explicitly conceptualizes the body embedded within its cultural and social contexts. Within this framework, culture-bound syndromes involving emotional disturbance and visceral dysregulation include *Hwa-byung* in Korea. *Hwa-byung* is characterized by anger-related affective and somatic manifestations, such as perceived injustice, resentment, and anger, accompanied by chest tightness, heat sensations, and a feeling of upward pressure. In TEAM, *Hwa-byung* is conceptualized as a state of psychological constraint that arises from disrupted circulation and regulation of Qi *(*Yoon et al., *2024**)*. Interventions that restore Qi circulation are used to re-establish the balance between mind and body. Notably, integrated mindfulness and Qigong training has proven effective for individuals with *Hwa-byung* and depressive disorders, enhancing both physical and psychological vitality (Yoon et al., 2025).

TEAM interventions are aimed at restoring harmonious flow. Qigong and tai chi are commonly classified as forms of “meditative movement” (Larkey et al., 2009). Meditation enhances attentional stability and nonreactivity, whereas both Qigong and tai chi combine breath, posture, and attentional control to build interoceptive awareness and regulate arousal (Osypiuk et al., 2018). Transient emotional states and chronic mood disorders are associated with changes in posture and movement. For instance, happiness is elicited by rising, upward, and rhythmic movement patterns, whereas sadness is elicited by downward, sinking patterns (Shafir et al., 2015). Minor, targeted modifications to the motor system, including the reversal of depression-associated postures, can shift negatively biased self-referential memory toward greater balance (Michalak et al., 2014) and may serve as a viable therapeutic target (Osypiuk et al., 2018).

Acupuncture is employed to modulate local and systemic dynamics through stimulation of specific points and channels (Hwang et al., 2021; Lee and Chae, 2021). By combining prior experience and expectations with sensory input, the brain constructs an individualized generative model that shapes how acupuncture is perceived (Kang et al., 2025). Furthermore, needle stimulation elicits *DeQi* sensation locally or at a distance from the insertion site, and the spatial properties of these sensations are thought to be related to therapeutic outcomes (Jung et al., 2016). Patterns of bodily sensation linked to fear heighten perceptual bias toward fearful faces (Jung et al., 2020). In contrast, sensations localized to the chest, but not to other body regions, can inhibit the perception of fearful faces (Jung et al., 2021).

Mindfulness is an intentional state of paying attention to present-moment internal experiences with a nonjudgmental attitude (Shapiro et al., 2006). It is a core mechanism across TEAM interventions, including meditation and Qigong, that emphasizes attention to and awareness of interoceptive sensations as a therapeutic process. Accordingly, TEAM can be understood as operating within a framework in which embodied emotions arise from direction, flow, and balance, and treatments aim to restore or recalibrate these dynamic patterns.

Clinical practice in acupuncture reflects these theoretical descriptions, illustrating emotion as an embodied state. Patients often present with palpitations or diffuse inner unease without primary cardiac pathology, accompanied by mild tremor, exaggerated startle, chronic nervousness, and anxiety. Within TEAM, this constellation is interpreted as Qi deficiency, and interventions that strengthen Qi (e.g., acupuncture, herbal formulas) often alleviate both somatic discomfort and affective instability in tandem. A different profile, characterized by chronic muscular tension in the temples, posterior neck, and occipital region, co-occurs with irritability, emotional lability, and anger, and is conceptualized as Qi stagnation. Similarly, patients with prolonged sleep deprivation commonly report dry eyes, nocturnal leg cramps, and heightened emotional sensitivity, corresponding to impaired blood nourishment and circulation. Across these patterns, emotional distress is experienced as a reproducible bodily condition rather than a purely mental event, aligning with interoceptive accounts in which shifts in visceral and somatic signals dynamically shape emotional experience.

## 3 Bridging perspectives: mapping emotions and mechanisms

### 3.1 Mapping emotions in the body

**Figure 1:**
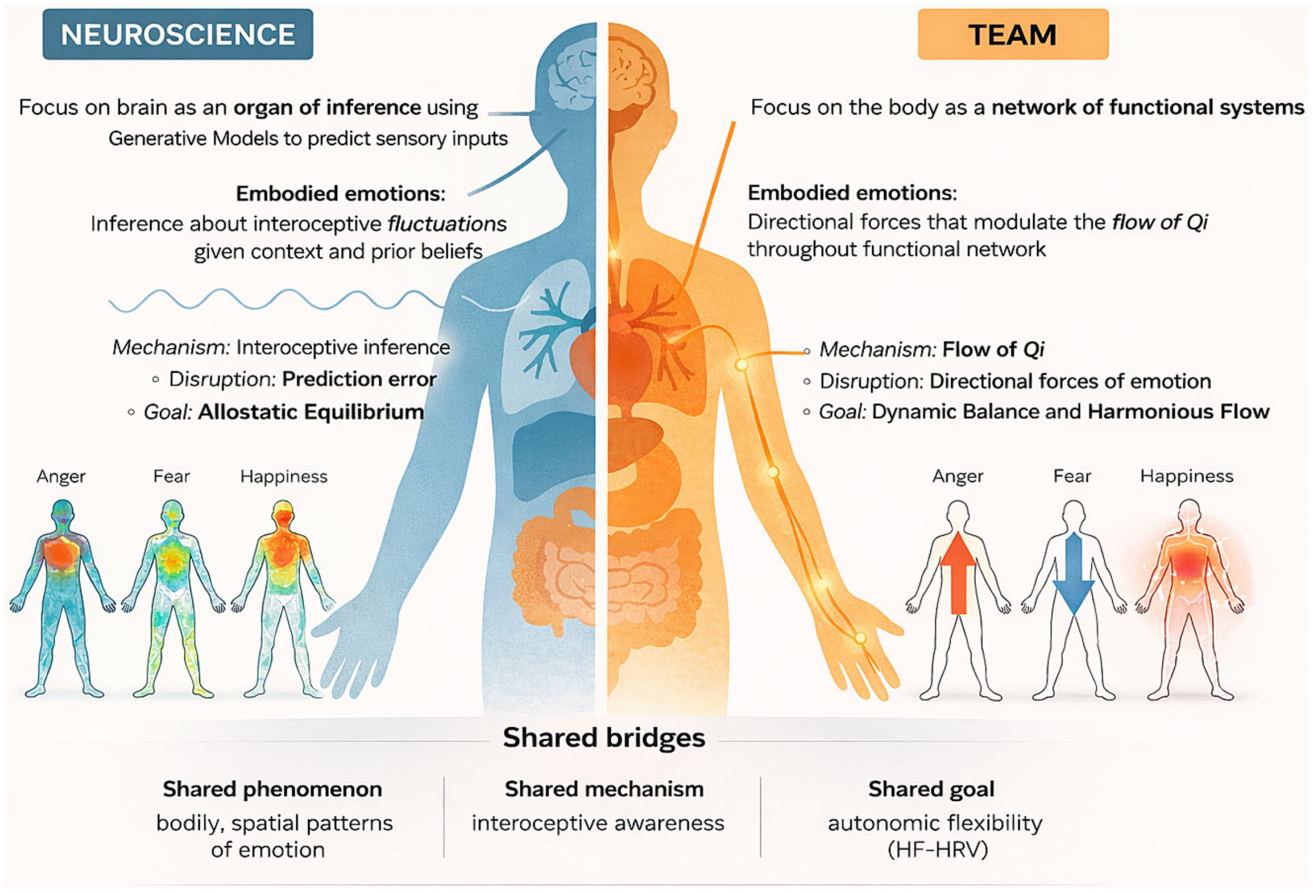
Bridging of neuroscience and TEAM models of embodied emotion. A combination of a neuroscience framework, focused on emotions arising from interoceptive inference and prediction error, and a TEAM framework, in which emotions act as directional forces shaping the flow of Qi through functional body networks. Both perspectives highlight spatial, embodied patterns of emotional experience. The three small heat-map figures on the left (anger, fear, happiness) are modified from Jung et al. (2017). Shared bridges emphasize convergence across models, including bodily patterning of emotion, mechanisms, and the goal of enhancing autonomic flexibility.

Accumulating phenomenological evidence suggests that emotions are spatially anchored in bodily experience. In a cross-cultural study, participants shaded body silhouettes to indicate where in the body they felt specific emotions, consistent and discriminable topographies emerged for happiness, sadness, fear, anger, and other states (Nummenmaa et al., 2014). For example, anxiety and fear were concentrated in the chest and abdomen, sadness emphasized sensations in the chest and limbs, anger highlighted the head and arms, whereas happiness showed widespread activation. These spatial patterns of emotion align with the directional framing of TEAM (anger as upward, fear as downward, joy as dispersion), highlighting a common phenomenon of how emotions are experienced in the body. These “body maps” suggest that emotion-specific bodily sensations may reflect distinct physiological configurations, and that affective categories track patterned interoceptive-perceptual signatures. Individual differences in emotional awareness correlate with the ability to experience interoceptive feelings (Herbert et al., 2011), and individuals with higher interoceptive accuracy are more likely to report stronger sensations in emotion-specific bodily locations (Jung et al., 2017) (Figure 1).

The topography of bodily sensations may thus serve as a fundamental index of emotional coherence and experience. Although such maps are subjective and probabilistic, they nevertheless indicate stable relationships between bodily sensations, categorical emotion, and conceptual labeling. A recent review suggested that bodily maps are reflections of physiological monitoring as well as expressive interfaces between brain, body, and conceptualization (Daikoku et al., 2025). Within a predictive framework, they can be interpreted as learned correspondences between interoceptive patterns and emotion concepts, shaped by culture and experience (Barrett, 2017). Notably, this perspective resonates with a longstanding clinical intuition in TEAM: emotions are embodied phenomena, with discernible “directions” and “weights” that can be felt, described, and modulated.

### 3.2 Mechanistic bridges: allostasis and autonomic flexibility

Allostasis, or the predictive regulation of internal states to meet anticipated demands, provides a useful unifying construct (Sterling, 2012). High-frequency heart rate variability (HF-HRV), a measure of autonomic flexibility that reflects parasympathetic control, has been associated with resilience and emotional regulation (Laborde et al., 2017; Porges, 2007). The emphasis of TEAM on dynamic balance aligns naturally with such indices, suggesting operational bridges between traditional constructs and contemporary biomarkers.

An example of this bridge is breathwork. Slow, prolonged exhalation augments vagal influence on the sinoatrial node, lowering heart rate and perceived arousal; subjectively, the Qi “settles.” Randomized trials and meta-analyses indicate that Qigong and tai chi are associated with improvements in HRV, mood, and stress outcomes, consistent with enhanced autonomic flexibility and heightened interoceptive clarity (Wang et al., 2010). Acupuncture has documented effects on limbic and brainstem networks and autonomic markers, although the operative mechanisms remain a matter of debate (Hui et al., 2007).

### 3.3 Recalibrating prediction error

Mindfulness, related body-based practices, and TEAM practices act on bodily awareness and predictive processing to support adaptive emotional regulation. Mindfulness enhances awareness of interoceptive signals and facilitates the accurate detection of prediction errors, and ultimately contributes to the development of more accurate predictive models that more closely align with reality (Kirk and Montague, 2015; Kirk et al., 2019). Individuals with depression often display emotionally biased prediction-error processing, characterized by attenuated reward prediction-error signals and heightened sensitivity to punishment- or risk-related prediction errors (Kumar et al., 2018; Rouhani and Niv, 2019). Mindfulness meditation increases activation of the posterior insula, a region associated with interoceptive processing, and modulates prediction-error signaling, suggesting that mindfulness training may support improved emotional regulation (Kirk and Montague, 2015; Kirk et al., 2019). Furthermore, because mindfulness fosters a receptive and nonjudgmental attitude toward experience, it is thought to reduce the precision of rigid priors and decrease maladaptive experiential avoidance (Arch and Craske, 2006; Farb et al., 2007). Collectively, these findings suggest that mindfulness may function as a dynamic mechanism of equilibrium, enabling continuous adjustment of priors via ongoing evaluation and present-moment evidence.

Mindfulness-based practices similarly improve interoceptive awareness and attenuate affective reactivity, plausibly by recalibrating the precision-weighting of interoceptive signals and fostering nonreactive appraisal (Khalsa et al., 2018; Allen et al., 2022). This mechanism parallels TEAM practices that cultivate attentional stability and interoceptive clarity through slow breathing, posture regulation, and meditative movement. Interventions may modify interoceptive processing through vagal neuromodulation, slow-breathing protocols that alter respiratory rate and depth, and awareness-based approaches such as mindfulness-based interventions (Weng et al., 2021).

While mindfulness emphasizes interoceptive awareness and the precision of bodily signals, other clinical approaches target prediction errors linked to emotions such as fear and threat. For example, interoceptive exposure reduces the precision of threat- and fear-related bodily priors by safely exposing patients to feared sensations. In a study on adolescents with chronic pain, those with high baseline fear showed larger pain reductions under interoceptive exposure compared to control conditions (Flack et al., 2018). Pain reprocessing therapy targets fear-related prediction errors by relearning pain beliefs and reducing catastrophizing. In a study on patients with chronic lower back pain, it produced significant pain relief relative to placebo and usual care, mediated by decreased fear and threat beliefs (Ashar et al., 2022). From the perspective of predictive processing, these practices may recalibrate interoceptive predictions and precision-weighting by updating beliefs about bodily signals and modulating autonomic set points (Seth and Friston, 2016; Allen et al., 2022).

## 4 Toward an integrative research program

Integrating TEAM and predictive neuroscience requires a rigorous understanding of the core constructs of each framework and an explicit acknowledgement of the differences between their categorical schemes. Recent conceptual analyses have also discussed potential correspondences between predictive mind frameworks, allostatic regulation, and traditional Chinese medicine, emphasizing functional analogies while acknowledging the epistemological differences between these traditions (Minelli, 2026). Facial expressions of fear enhance sensory acquisition, whereas disgust expressions restrict it. Facial expressions are not merely arbitrary patterns used for social communication; rather, they may originate from physiological adjustments that regulate sensory access to the physical world (Susskind et al., 2008). Directional metaphors in TEAM can be used to formulate testable hypotheses about spatial and physiological patterns. For example, anger (construed as “rising”) may correspond to cephalic vasodilation, elevated facial temperature, laryngeal tension, and upward postural sway; fear (interpreted as “descending”) may involve activation of lower abdominal muscles, diaphragmatic restriction, and downward sway. Such patterns can be quantified using methods such as posturography (to assess sway), respiratory inductance plethysmography (to measure breathing dynamics), inertial measurement units (to capture movement), and thermal imaging (to track changes in facial temperature). From a predictive neuroscience perspective that emphasizes the precision-weighting of sensory prediction errors, TEAM practices may influence interoceptive precision by either enhancing signal clarity (e.g., through slow, attentive breathing) or attenuating maladaptive gain (e.g., down-weighting hypervigilant priors). Pre/post experimental designs could include heartbeat discrimination tasks, respiratory interoceptive tasks, insula connectivity assessments, and symptom outcomes to evaluate these systems.

A recent study showed that targeting interoception can alter symptom trajectories. Ecological momentary assessment demonstrated that interoceptive accuracy, awareness, and sensibility fluctuate substantially within individuals over days, underscoring the need for repeated measurement when evaluating interoception-focused treatments (Holler et al., 2021). Thus, we recommend repeated real-time sampling of participants’ experiences in their natural environments to capture interoceptive changes during TEAM practices such as breathwork, Qigong, or acupuncture. This approach can reveal immediate physiological and affective responses, track temporal dynamics, support causal inference about treatment-related shifts, identify moderators and mediators of response and, ultimately, inform the optimization and personalization of interventions (Shiffman et al., 2008).

Building on these measurement strategies, integrative trials can apply them to clinical conditions where interoception plays a central role. Chronic pain, depression, and anxiety represent particularly promising contexts. Factorial designs could test whether combining breathwork and acupuncture (e.g., by jointly adjusting autonomic tone and interoceptive predictions) produces greater benefit than either treatment alone. Baseline interoceptive profiles may serve as moderators, helping to identify subgroups most likely to respond. The TEAM construct of balance can be indexed through multimodal biomarkers such as HF-HRV (parasympathetic flexibility), respiratory sinus arrhythmia, baroreflex sensitivity, cardiorespiratory coupling, gastric–brain phase synchronization, and validated self-reports of interoceptive awareness (Laborde et al., 2017). Composite indices derived from these measures may provide useful indicators of systemic regulation or “harmonious flow.”

At the same time, conceptual translation carries risks of conflation. Qi cannot be reduced to a single physiological parameter such as vagal tone or heart rate variability; rather, it functions as a broader conceptual framework describing systemic patterns of regulation across the body. TEAM itself is not a single, uniform system but a collection of different schools and interpretive traditions. Expectancy and nonspecific factors also influence outcomes in mind–body interventions, making rigorous controls, blinding where feasible, and preregistration essential. Predictive processing theories are likewise evolving, so care is required to avoid overextending metaphors or presenting untested mechanisms as established facts. The correspondences we propose between TEAM constructs and contemporary biomarkers or computational concepts should therefore be understood as heuristic mapping hypotheses rather than claims of direct equivalence. Their value lies in generating cross-constraints on theory and experiment, allowing each framework to refine, challenge, and inform the other. Finally, we emphasize that TEAM concepts have emerged within specific historical, cosmological, and sociocultural contexts; a genuinely pluralistic approach therefore treats these traditions as interlocutors rather than attempting to subsume one framework within the other.

## 5 Conclusion

An embodied predictive view of emotion situates cognition within a broader system of metabolism, autonomic regulation, and interoceptive inference. In this respect, contemporary neuroscience and TEAM converge in emphasizing that emotional experience is grounded in dynamic patterns of bodily regulation. Rather than proposing a direct theoretical equivalence, this perspective highlights points of convergence between predictive neuroscience and TEAM as heuristic bridges for interdisciplinary research. Concepts such as interoception, autonomic flexibility, and allostasis may provide operational entry points for investigating traditional ideas of balance, direction, and flow. Future studies integrating physiological measurement, computational models, and clinical research on mind–body interventions may help clarify these relationships, particularly in conditions where interoceptive processes play a central role. Such dialogue may contribute to the development of a more comprehensive science of embodied emotion that meaningfully bridges neuroscience and traditional medical knowledge.

## Data Availability

The original contributions presented in the study are included in the article/supplementary material, further inquiries can be directed to the corresponding author.
